# Palliative care is a viable option for frail elderly patients with neurocognitive disorders admitted for hip fractures

**DOI:** 10.1186/s12891-024-07739-w

**Published:** 2024-08-10

**Authors:** Justine Boulet, Etienne L. Belzile, Norbert Dion, Chantal Morency, Mélanie Bérubé, Alexandra Tremblay, Stéphane Pelet

**Affiliations:** 1 Department of Surgery, Division of Orthopedic Surgery, CHU de Quebec-Universite Laval, Quebec City, QC Canada; 2 Department of Surgery, Faculty of Medicine, Laval University, Quebec City, QC Canada; 3 Palliative Care Unit, Department of Medicine, CHU de Quebec-Universite Laval, Quebec City, QC Canada; 4 Population Health and Optimal Practices Research Unit, CHU de Quebec-Universite Laval Research Centre, Quebec City, QC Canada; 5 Faculty of Nursing, Laval University, Quebec City, QC Canada

**Keywords:** Pallaitive care, Hip fracture, Neurocognitive disorder

## Abstract

**Importance:**

Most patients presenting with a hip fracture regardless of their comorbidities are surgically treated. A growing body of research states that a certain type of elderly patient could benefit more from a palliative approach.

**Objective:**

Identify the patient who would benefit most from a palliative care approach instead of a surgery.

**Design:**

Exploratory-matched retrospective cohort study between 2015 and 2021.

**Setting:**

Single Level 1 Trauma Center.

**Participants:**

There were 2240 hip fracture patients admitted to our institution between 2015 and 2021. Patients over 65 years old with intertrochanteric or femoral neck fractures could be included. A total of 129 patients opted for palliative care (Palliative Group = PG). This cohort was compared to a matched cohort (for age, sex and fracture type) who underwent surgery but died within three months of the procedure (Surgery Deceased Group = SDG) and another matched cohort who survived more than three months (Surgery Alive Group = SAG) following surgery.

**Main outcomes and measures:**

Medical charts were reviewed for patient demographics, autonomy level, level of care, neurocognitive disorders (NCD), fracture type, in-hospital data and outpatient death within three months of admission. Analysis was performed through univariate and multivariate models with SAS OnDemand for Academics (alpha 0.05).

**Results:**

Patients in the PG (*n* = 129) were 88.2 ± 7.2 years old, 71.3% were females, and 61.2% had a femoral neck fracture. Patients in the SDG (*n* = 95) and SAG (*n* = 107) were well matched. The PG differed from the SDG (*n* = 95) and SAG (*n* = 107) regarding NCD (85.3% vs. 57.9% vs. 36.4%, *p* < 0.01) and the presence of Behavioral and psychological symptoms of dementia (BPSD) (19.4% vs. 5.3% vs. 3.7%, *p* < 0.01). There were more known heart failure (24.2% vs. 16.3%, *p* < 0.01) and Chronic Obstructive Pulmonary Disease (COPD) in the SDG group than in the PG group (26.6 vs. 14.7%, *p* = 0.02). Patients in the SAG have a significant lower rate of NCD (OR 2,7 (95%CI 1,5–5,0)), heart failure (OR 5,7 (95%CI 1,9–16,4)) and COPD (OR 2,8 (95%CI 1,2–6,3)) than other groups. Prefracture mobility, autonomy and living situation significantly differed between the groups. Median survival was six days in PG and 17 days in SDG. All groups lost autonomy and mobility. There were more complications in the SDG group than in the PG group. The end-of-care trajectory was death or hospice for most patients in the PG and SDG groups. More than 30% of the SAG group could not return home at discharge.

**Conclusion:**

The presence of an NCD and diminished prefracture autonomy strongly support counseling for palliative care. The high rate of complications when surgery is proposed for frail patients with multiple comorbidities suggests that the concept of palliative surgery needs to be revisited.

**Supplementary Information:**

The online version contains supplementary material available at 10.1186/s12891-024-07739-w.

## Introduction

Hip fractures are common among the elderly population, with 147 hip fractures per 100,000 inhabitants in Canada in 2014–2015. Projections estimate 6.3 million hip fractures worldwide in 2050 [1]. Despite prompt surgical treatment, high mortality rates of 15% at 30 days and 18% at one year are reported and rise to 33.5% at 30 days and 48.8% at one year with nonoperative treatment [2, 3]. Several factors have been identified as risk factors for early mortality or morbidity: high ASA score, neurocognitive disorder, low mobility and autonomy level, male gender, older age, and longer surgical delays [3–5].

Frail patients with a surgically treated hip fracture require very important postoperative resources and put increased pressure on the caregivers and the family. Even with a multidisciplinary approach, a large proportion of patients will not recover their previous autonomy level [6–11]. Transfers to more involved care facilities are often necessary, delaying hospital discharge. Based on these findings, the use of palliative care treatment has attracted growing interest, mainly for frail patients with neurocognitive disorders, low autonomy levels and very severe comorbidities [6, 12–15]. The published evidence is still poor and is mainly based on small retrospective series [13–15]. There is little available data to select the best patient for palliative care, nor how to deliver this care efficaciously.

In the absence of clinical guidelines or societal recommendations, the role of palliative management remains largely unknown in the current healthcare system. This study aims to identify which patients would benefit from palliative care rather than surgery for a hip fracture.

## Methods

An exploratory retrospective matched cohort study including all patients over 65 years old admitted for a hip fracture (femoral neck and intertrochanteric) between 2015 and 2021 was conducted in a level one trauma center. The study received full ERB approval and was performed in accordance with the Helsinki declaration and STROBE statements. Patients with a subtrochanteric fracture, periprosthetic fracture, or pathologic fracture were excluded.

Three cohorts were constituted to answer the goal of this study:

1. A control cohort including patients directly referred to the palliative care unit without undergoing surgery (Palliative Group - PG).
2. A cohort including surgically treated patients who passed away within three months after the surgery (Surgical Deceased Group – SDG).
3. A cohort including surgically treated patients who were still alive three months after the surgery (Surgical Alive Group – SAG).

The second and third cohorts were matched to the control cohort for age, sex and fracture type. A three-month threshold to separate deceased and living patients was based on many previous studies illustrating the long-term follow-up of hip patients, mainly exploring mortality rates [12–15].

Hospital medical charts were reviewed by two independent orthopedic registrars not involved in the treatment of the patients. PG patients were all admitted by the palliative care team, and specific prospective data were collected by the general physician in charge to assess the quality of management.

Exploratory variables of interest are divided into three categories: (a) patient demographics (age, sex, medical comorbidities, Charlson score, autonomy level, level of care, neurocognitive disorders (NCD), fracture type); (b) in-hospital data (length of stay, surgical data for the SDG and SAG subgroup, complications including death, discharge location); and (c) outpatient death within 3 months of admission.

The diagnosis of NCD is based on a previous diagnosis available at admission or with a score less than 24 on the Mini Mental State Examination (MMSE) [16] or less than 20 on the Montreal Cognitive Assessment (MoCA) [6].

The level of care at admission is based on a preemptively completed notarized document and consists of four levels, ranging from A (full code) to D (palliative care) [7] (Appendix 1).

### Palliative care management

The decision not to operate was made through a shared decision-making process involving the patient and family, especially if there was any sign of neurocognitive disorders. A palliative care physician was also involved. Institutionalized patients were returned to their hospice if adequate palliative care could be offered.

If admitted, care was focused on comfort. Patients’ needs for analgesia were assessed by trained nursing staff using the PACSLAC scale [8], and regular analgesics were prescribed by the palliative care specialist. There was no plan for early mobilization. If a complication arose, it was not medically treated. We observed three possible scenarios:

- Some patients became rapidly lethargic. Care was then focused on end-of-life symptoms, and only low doses of regular narcotics were usually necessary for pain management.
- Some stabilized after a few days and developed a failure to thrive syndrome (“Syndrome de glissement”, a French geriatric concept) characterized by anhedonia, anorexia, withdrawal and opposition [9], which led to death. Care was focused on pain management as well as Behavioral and psychological symptoms of dementia (BPSD)management. When present, small dosages of methotrimeprazine, a first-generation antipsychotic, were used.
- Others were more energetic and vigorous despite a palliative care approach. After a while these patients usually ate their meals in the chair, and some of them even walked with some assistance. They did not require higher doses of narcotics. Eventually, these patients would be discharged to a hospice or an assisted living home.

### Surgical management

After an adequate medical evaluation, most patients underwent surgery within 48 h of admission [10] with adequate fixation or arthroplasty under general or spinal anesthesia. Patients admitted with an unstable medical condition were first stabilized with the orthogeriatric team, then surgery was performed. No patient in this group was considered for palliative care after admission. Postoperatively, patients were submitted, if possible, to an early mobilization protocol. Patients were followed by physical therapists as well as by a hospitalist who managed their medical complications. Functional progress was monitored, and the ultimate trajectory of patients was discussed at multidisciplinary meetings.

### Statistical analysis

Descriptive statistics were performed to compare PG to SDG first and then to SAG and SAG to SDG. SDG is considered the group where palliative care could have been considered, and SAG represents the patients who will benefit from surgery. Patients in PG constituted the control cohort (no exclusion) and all patients perfectly matching for age, sex and fracture type during the study period in the two other groups were included. Values are expressed as the means or medians with 95% CIs or standard deviations or percentages with ranges. Univariate analyses were performed to detect differences between groups with a Chi square test or Fisher’s test for categorical data and Student’s t test for parametric values. Multivariate analyses was performed to determine the significant factors that suggest patients in the SDG would be better served by PG (binomial logistic regression model with Wald confidence intervals). All data were treated with SAS OnDemand for academics (2023 SAS Institute, Cary, NC). An alpha error of 5% was arbitrarily set.

## Results

During the study period, 2240 hip fractures were admitted to our institution. Palliative care management was emerging, and 129 patients were gradually included in the PG (2 in 2015, 8 in 2016, 6 in 2017, 20 in 2018, 17 in 2019, 30 in 2020 and 46 in 2021). One hundred and seventy-nine surgically treated patients died during the first 3 months, and we could perfectly match 91 patients for age, sex and fracture type (SDG). For SAG, we extracted data from 300 patients still alive on the day of extraction and were able to select 107 of them.

Table 1 illustrates the demographic characteristics of the three cohorts. The mean age was 87.5 years old, most patients were female (72%), and femoral neck fractures represented 61% of all fractures.

**Table 1:** Demographic characteristics of the Study Population

	Palliative group (PG)	Surgical Deceased Group (SDG)	Surgical Alive Group (SAG)	*p* (PG vs. SDG)	*p* (PG vs. SAG)	*p* (SDG vs. SAG)
	*n* = 129	*n* = 91	*n* = 107			
Mean Age *	88,3 (87,0–89,5)	87,9 (86,5–89,4)	86,3 (85,1–87,5)	0.74	0.13	0.27
**Sex** ^**&**^				0.98	0.67	0.71
Female	92 (71,3%)	65 (71,4%)	79 (73,8%)			
Male	37 (28,7%)	26 (28,6%)	28 (26,2%)			
**Fracture type** ^**&**^				0.96	0.82	0.81
Femoral neck	79 (61,2%)	56 (61,5%)	64 (59,8%)			
Intertrochanteric	50 (38,8%)	35 (38,5%)	43 (40,2%)			
**NCD** ^**&**^	110 (85,3%)	53 (58,2%)	39 (36,4%)	**< 0**,**01**	**< 0**,**01**	**< 0**,**01**
**BPSD** ^**&**^	25 (19,4%)	4 (4,4%)	4 (3,7%)	**< 0**,**01**	**< 0**,**01**	1
Charlson index *	2,8 (2,4 − 3,2)	2,4 (2,1–2,8)	1,4 (1,1–1,6)	0.18	**< 0**,**01**	**< 0**,**01**
**Medical comorbidities** ^**&**^						
Coronary artery disease	38 (29,5%)	39 (42,9%)	44 (41,1%)	**0.04**	0.06	0.81
Peripheral artery disease	36 (27,9%)	17 (18,7%)	17 (15,9%)	0.12	**0.02**	0.6
Arrythmia	44 (34,1%)	36 (40%)	29 (27,1%)	0.4	0.25	0.06
Chronic obstructive pulmonary disease	19 (14,7%)	24 (26,7%)	12 (11,2%)	**0.02**	0.43	**< 0**,**01**
Heart Failure	21 (16,3%)	22 (24,2%)	5 (4,7%)	0.15	**< 0**,**01**	**< 0**,**01**
Diabetes mellitus	16 (12,4%)	20 (22%)	25 (23,4%)	0.06	**0.03**	0.82
Chronic renal insufficiency	41 (31,8%)	36 (40%)	29 (27,1%)	0.23	0.43	0.06
Stroke	27 (21%)	17 (16,8%)	16 (15%)	0.56	0.24	0.59
Active neoplasia	15 (11,6%)	5 (5,5%)	2 (1,9%)	0.12	**< 0**,**01**	0.17
**Level of care at admission** ^**&**^				0.13	**< 0**,**01**	**0.01**
A	3 (2,3%)	1 (1,1%)	7 (6,5%)			
B	33 (25,6%)	31 (34,4%)	35 (32,7%)			
C	42 (32,6%)	25 (27,8%)	18 (16,8%)			
D	17 (13,2%)	4 (4,4%)	0 (0%)			
Unknown	34 (26,4%)	29 (32,2%)	47 (43,9%)			

* Values expressed as means (95% CI) ^&^ Values expressed as N (%)

The prevalence of NCD was significantly higher in the PG than in the two other groups and even higher in the SDG than in the SAG. The prevalence was still important in the SAG (36.4%). BPSD represented the more debilitating form of NCD and were present in 19.2% of the PG (4.4% in SDG and 3.7% in SAG).

Patients in the PG and SDG presented at admission with more comorbidities than those who ‘’succeeded’’ surgical treatment (*p* < 0.01). The main interesting differences were a higher rate of heart failure and COPD in the SDG compared to the two other groups and a higher number of active neoplasia in the PG. The level of care at admission reflected the need to seriously think about palliative care with a nonsignificant difference between the PG and SDG patients (mainly level C and D when known), when this difference was significant with SAG patients (nearly half of them had no level established at admission, and most were at level A and B). The absence of a level of care was frequent in healthy patients (no previous discussion had been initiated with healthcare professionals).

Patients with NCD (OR 2,7 (95%CI 1,5–5,0)), heart failure (OR 5,7 (95%CI 1,9–16,4)) and COPD (OR 2,8 (95%CI 1,2–6,3)) have a higher risk in the SDG and treatment palliatively might be considered.

We observed a very important difference in autonomy status at admission (Table 2). Patients in the PG and SDG were significantly frailer than those in the SAG (*p* < 0.01), with 90% in the PG and 78% in the SDG fully dependent, corresponding to a grade 7 or more on the Frailty Index (FI-CGA) [11]. Most patients in the PG (81.4%) were living in healthcare facilities with services when sustaining the hip fracture, compared to 41.1% and 65.4% living at home for the SDG and SAG, respectively (*p* < 0.01).

**Table 2:** Autonomy status at Admission

	Palliative group (PG)	Surgical Deceased Group (SDG)	Surgical Alive Group (SAG)	*p* (PG vs. SDG)	*p* (PG vs. SAG)	*p* (SDG vs. SAG)
	*n* = 129	*n* = 91	*n* = 107			
**Mobility**				**0.05**	**< 0,01**	**< 0,01**
No walking aid	36 (29,9%)	41 (45,1%)	61 (57%)			
Cane	2 (1,6%)	2 (2,2%)	13 (5,5%)			
Walker	63 (48,8%)	38 (41,8%)	28 (26,2%)			
Non ambulatory	9 (7%)	2 (2,2%)	0 (0%)			
Unknown	19 (14,7%)	8 (8,8%)	5 (4,7%)			
**Autonomy level**				**0.01**	**< 0**,**01**	**< 0**,**01**
Independent	13 (10,1%)	20 (22%)	79 (73,8%)			
Dependent	116 (89,9%)	71 (78%)	27 (25,2%)			
Unknown	0 (0%)	0 (0%)	1 (1%)			
**Living situation**				**< 0**,**01**	**< 0**,**01**	**< 0**,**01**
Home or Senior home without services	24 (18,6%)	37 (41,1%)	70 (65,4%)			
Senior home with services	67 (51,9%)	31 (34,4%)	24 (22,4%)			
Hospice	38 (29,5%)	20 (22,2%)	11 (10,3%)			
Unknown	0 (0%)	2 (2,2%)	2 (1,9%)			

Values expressed as N (%)

During the hospital stay, we observed a major difference in the complication rates between SDG compared to PG and SAG: these patients suffered more severe complications, such as pneumonia, acute coronary syndrome, acute renal failure and cardiac deficiency with acute pulmonary edema, most of which led to death. Delirium was equally present in the three cohorts, between 20 and 30%. PG presented fewer thromboembolic events, and we observed a low rate of pressure sores in all groups. Interestingly, the hospital stay was significantly lower in the PG group (9.6 days vs. 14.7 days and 16.4 days, *p* < 0.01) (Table 3).

**Table 3:** In-Hospital evolution

	Palliative group (PG)	Surgical Deceased Group (SDG)	Surgical Alive Group (SAG)	*p* (PG vs. SDG)	*p* (PG vs. SAG)	*p* (SDG vs. SAG)
	*n* = 129	*n* = 91	*n* = 107			
Type of surgery*						0.74
Cephalomedullary nail		21 (23%)	24 (22,4%)			
Dynamic Hip Screw		16 (17,6%)	19 (17,8%)			
Cannulated screws		5 (5,5%)	10 (9,3%)			
Hemiarthroplasty		49 (53,9%)	54 (50,5%)			
**Time to surgery (days)** ^**&**^		1,6 (2,4; 0–16)	1,5 (2,1;0–17)			0.77
**Hemoglobin at admission (g/l)** ^**&**^	120,6 (17,8;67–165)	108,6 (18,4; 56–144)	118,3 (20,1;69–156)	**< 0**,**01**	0.37	**< 0**,**01**
**Length of stay (days)** ^**&**^	9,6 (16,7;0-143)	14,7 (12,2; 1–47)	16,4 (16,1; 3–99)	**0.01**	**< 0**,**01**	0.43
Complications*						
Pneumonia	22 (17,1%)	29 (31,9%)	3 (2,8%)	**0.01**	**< 0**,**01**	**< 0**,**01**
Acute coronary syndrome	4 (3,1%)	17 (18,7%)	5 (4,7%)	**< 0**,**01**	0.22	**< 0**,**01**
Acute renal failure	9 (7%)	24 (27%)	9 (8,4%)	**< 0**,**01**	0.68	**< 0**,**01**
Cardiac deficiency	9 (7%)	27 (27,9%)	2 (1,9%)	**< 0**,**01**	0.05	**< 0**,**01**
Delirium	29 (22,7%)	28 (30,8%)	22 (20,6%)	0.18	0.7	0.1
Urinary Tract Infection	7 (5,4%)	10 (11%)	8 (7,5%)	0.13	0.52	0.39
Thromboembolic event	1 (0,8%)	10 (11%)	5 (4,7%)	**< 0**,**01**	0.06	0.1
Pressure sore	4 (3,1%)	6 (6,6%)	2 (1,9%)	0.23	0.28	0.09
Surgical Site Infection		2 (2,3%)	2 (1,9%)			0.85
In-hospital death*	99 (76,7%)	64 (70,3%)	0 (0%)	0.28	**< 0**,**01**	**< 0**,**01**

*Values expressed as N (%) ^&^Values expressed as means (SD; range)

Most patients in the PG and SDG never left the hospital (Table 4). Most patients in both surgical groups moved to a residence with more services than at admission. Only 31.8% of the patients in the SAG could return home or in a senior home. All patients lost autonomy and dependence after the episode of care. Patients in the SDG had autonomy levels at discharge similar to those of PG patients, corresponding to level 8 on the Frailty Scale Index.

**Table 4:** Autonomy status at Discharge

	Palliative group (PG)	Surgical Deceased Group (SDG)	Surgical Alive Group (SAG)	*p* (PG vs. SDG)	*p* (PG vs. SAG)	*p* (SDG vs. SAG)
	*n* = 129	*n* = 91	*n* = 107			
**Mobility**				**< 0,01**	**< 0,01**	**< 0,01**
No walking aid	1 (0,8%)	2 (2,2%)	2 (1,9%)			
Cane	0 (0%)	0 (0%)	3 (2,8%)			
Walker	2 (1,6%)	19 (20,9%)	90 (84,1%)			
Non ambulatory	125 (96,8%)	64 (70,3%)	4 (3,7%)			
Unknown	1 (0,8%)	6 (6,6%)	8 (7,5%)			
**Autonomy level**				0.23	**< 0**,**01**	**< 0**,**01**
Independent	0 (0%)	1 (1,1%)%)	17 (15,9%)			
Dependent	129 (100%)	90 (98,9%)	89 (83,2%)			
Unknown	0 (0%)	0 (0%)	1 (0,9%)			
**Trajectory at discharge**						
Home or senior home without services	0 (0%)	2 (2,2%)	34 (31,8%)	**0.04**	**< 0**,**01**	**< 0**,**01**
Senior home with services	3 (2,3%)	3 (3,3%)	11 (10,3%)			
Hospice	22 (17,1%)	11 (12,1%)	13 (12,2%)			
Rehabilitation center	0 (0%)	4 (4,4%)	29 (27,1%)			
Regional hospital	0 (0%)	0 (0%)	17 (15,9%)			
Palliative Care Unit	5 (3,9%)	7 (7,7%)	0 (0%)			
Death	99 (76,7%)	64 (70,3%)	0 (0%)			

Values expressed as N (%)

The median survivorship was used to best compare this issue between PG and SDG, as four patients in the PG were not dead at the time of the review. The median survivorship was 6 days in the PG (SD 195.9, range 1-1424) and 16 days in the SDG (SD 22.4, range 1–88). Patients in the SAG had a significantly better survivorship (median 1652, SD 649, range 176–2900), and only two (1.9%) died within the first year after surgery. When looking at the evolution during the hospital stay between PG and SDG, mortality was 56.6% vs. 36% at one week, 79% vs. 61% at two weeks, 85.3% vs. 72.8% at one month, and 91% vs. 100% at three months.

## Discussion

This study aimed to identify which of the frail patients presenting with a hip fracture would benefit the most from palliative rather than surgical treatment. The role of palliative nonoperative management in hip fracture care seems to have a place of choice for a subgroup of patients in the current healthcare system. We found that patients with neurocognitive disorders, an altered pre-fracture functional status, cardio-pulmonary comorbidities or multiple medical comorbidities would benefit the most from a palliative care approach rather than from surgery. The prevalence of NCDs and BPSD, which results in a more severe form of dementia, was higher in the PG and SDG groups. When looking at the patient’s living arrangement and level of independence pre-fracture, we realized that in both PG and SDG, patients had already experienced an altered autonomy status: 29.5% and 22.3% of patients would already live in a long-term care facility, and 89.9% and 76.8% were deemed dependent. COPD, HF and a higher Charlson Index were more prevalent in the SDG than in the other two cohorts. Patients with more medical comorbidities, especially COPD and HF, are not good candidates for surgical treatment, as their mortality rate is as high as in palliative care but with many more complications.

Dementia, as an independent risk factor, was already found to increase mortality and to negatively affect the ability to recover walking after hip fracture surgery in a systematic review and meta-analysis of 35 633 patients [12]. Patients with NCDs are not good candidates for rehabilitation and often become more dependent than their pre-fracture state, requiring hospice care^14^. The quality of life of these patients is profoundly altered, as they often do not recover the ability to walk, even with a walking aid. Indeed, 67.4% of patients in the SDG group were non-ambulatory at the end of their care trajectory. They also frequently need to be relocated if their original place of residence cannot provide enough help with ADLs.

When we look at the origin of our patients and their level of independence pre-fracture, in both PG and SDG patients had already experienced an altered autonomy status: 29.5% and 22.3% of patients would already live in a long-term care facility, and 89.9% and 76.8% were deemed dependent. It is already known that poor autonomy and mobility status pre-fracture are associated with a poor prognosis after surgery, as it increases by 18 times the chance of not being able to recover a basic ambulatory status [14]. Our results show that patients in the SDG group were less independent and mobile than those in the SAG group, resulting in 67% in-hospital deaths. Even with neuro-cognitively intact patients who were independent and had no walking aid, which were more prevalent in the SAG, only 31.2% of these patients were able to return to their initial home without services, while 15.1% remained independent and 84.1% needed a walker to be ambulatory.

Overall, higher Charlson index, COPD, and HF were more prevalent in the SDG than in the other two cohorts, thus associating pulmonary disease and more terminal cardiac disease as well as multiple comorbidities with a worse prognosis for hip fracture surgery [3]. Diabetes, PAD, and CAD did not seem to have influence, as they were more frequent in the SAG.

Mortality associated with nonoperative treatment of a hip fracture was initially found to be approximately 33.5% at one month and 48.8% at one year [2]. However, these numbers are associated with nonoperative treatment, such as early mobilization or bed rest with traction, ensuring that the patient stays alive with investigation and treatment of complications [2]. Even with prompt surgical treatment, 95 patients over five years in our institution died within three months of their surgery, on average 26.6 days after surgery. Moreover, this group had a mortality rate of 82% at one month. These patients suffered from many systemic complications and mostly died in-hospital instead of at home or in an end-of-life care unit.

When considering palliative care treatment for hip fracture with frail patients, the literature is scarce. However, recently, new insights have begun to emerge on this delicate subject. A group from the Netherlands [15] reported on a consecutive cohort of 91 frail patients managed nonoperatively; they found a 30-day mortality of 87%. Another group from the Netherlands [17] also published the results of a multicenter cohort study where 88 institutionalized patients (or their family) opted for nonoperative management for their hip fractures, and 84 opted for operative treatment. Interestingly, their nonoperative group also had a high cumulative mortality rate of 75% at 2 weeks and 83% at 30 days. Of note, 51% of the patients who died in the nonoperative group received palliative sedation (as found in the supplementary online content), which may represent a different reality than ours.

There are many barriers that prevent surgeons and clinicians from offering palliative care to frail patients with hip fractures and their families. Hip fracture surgery is often regarded as a palliative surgery, and the role of palliative care is somewhat unknown [18]. Our early experience with these 129 patients was positive. Pain is usually managed with low-dose narcotics and regular acetaminophen; the nursing staff is trained to detect signs of pain in nonverbal or noncommunicating patients. A palliative approach is different than nonoperative management for these patients: we do not submit them to an early mobilization protocol.

Although the goal of this paper was not to demonstrate the efficaciousness of palliative care, all patients in both groups should be considered for palliative care. Many surgeons are refractory to proposing palliative care after a hip fracture for many reasons, mainly thinking about less complications and less pain [18]. Patients in the SDG in this series may have the same fatal prognosis as patients in the PG, and with more surgical complications. All patients should be considered for palliative care after admission and palliative care concepts are certainly worthwhile to revisit.

As other authors published recently, we also found similar high cumulative mortality at 2 weeks (79%) and at 30 days (85.3%). These results certainly impact the way we approach hip fractures in frail patients, especially those with NCD. We can now discuss these results with the patients and their families to give them more insightful information in a shared decision-making process. As seen with the increasing number of such patients placed in the palliative group, from 2015 to 2021, giving more information to patients and their families enables them to be involved in the final decision while respecting their beliefs. Recent data [19] from a retrospective study also showed that including a preoperative comprehensive geriatric assessment led to significantly more patients and their families electing for nonsurgical management of their hip fracture.

The strength of our study is to be the first of its kind to compare palliative care to surgical treatment for hip fracture in the geriatric population in North America, also looking at patient’s outcome with palliative care for hip fracture. However, our population is not representative of all communities and results should be cautiously interpreted where the social setting is different. A recent report from authors in Alberta (Canada) demonstrates the reflection on the use of palliative care in different settings than our [20]. Our palliative group cohort was composed of 129 patients, which increases the power of our study, as it is a large group.

However, there are limitations to our study. Data recovered from written medical records were variable in quality, leading to measurement bias. Some patients had missing data on previous ambulatory status, autonomy level and/or living situation; hence, conclusions about the autonomy profile of the cohorts may not entirely reflect reality. For medical comorbidities, chronic diseases were gathered under the same label. However, there is a gradation in the severity of these diseases. As an example, first stage COPD versus O_2_-dependent COPD were just recorded as “COPD”. Conclusions regarding medical comorbidities need to be drawn carefully, as they can be critical pieces of information to improve care for hip fracture patients. Further studies should evaluate these variables prospectively as they could be critical knowledge to improve decision making after hip fractures.

## Conclusion

Palliative care for hip fracture with a multidisciplinary approach with palliative care specialists can be appropriate for geriatric patients presenting with NCD, diminished autonomy, and a non-ambulatory status. Palliative care could also be considered for patients with multiple comorbidities who are already very ill, as they might die briefly after surgery while suffering more complications. Further research should focus on developing decision tools including this information to help the surgeon to propose palliative care to the right patients. More research needs to focus on the efficaciousness of palliative care, including optimizing pain strategies.

### Electronic supplementary material

Below is the link to the electronic supplementary material.

Appendix 1

## Data Availability

The datasets used and/or analysed during the current study are available from the corresponding author on reasonable request.
