# Supplementary material for: Palliative care is a viable option for frail elderly patients with neurocognitive disorders admitted for hip fractures
Source: BMC Musculoskelet Disord. 2024 Aug 10;25:635. doi: 10.1186/s12891-024-07739-w (PMC11316310; doi:10.1186/s12891-024-07739-w)
Supplement: Supplementary file 1 — Appendix 1 [file 12891_2024_7739_MOESM1_ESM.pdf]

Santé
et Services sociaux
Québec

DT9262

LEVELS OF CARE AND CARDIOPULMONARY RESUSCITATION

The goals of care below are indicative and are intended to orient medically appropriate care.

Institution name

Last name of user

First name

File number | Date of birth Year Month Day

Sex ☐ M ☐ F | Health insurance number

Revise using a new form following any change in health status or at the request of the user or his/her representative.

Capacity to discuss levels of care

☐ Competent ☐ Incompetent: ☐ Homologated mandate ☐ Public/private curator; Name: ________________

☐ Minor under 14 years old Name of tutor, relationship with user: ________________

Previous advance wishes: ☐ None available ☐ Prior level of care form ☐ Advance medical directive ☐ Living will or other

Levels of care: check and provide details in the box below *(Explanatory notes on the reverse side)*

☐ Goal A: Prolong life with all necessary care

☐ Goal B: Prolong life with some limitations to care

☐ Goal C: Ensure comfort as a priority over prolonging life

☐ Goal D: Ensure comfort without prolonging life

Give details on specific interventions in the box below, as needed.

e.g., hemodialysis, blood transfusion, nutritional support (enteral or parenteral), preventive care, etc.

Cardiopulmonary resuscitation (CPR): check and provide details in the box below *(Explanatory notes on the reverse side)*

**Cardiac (circulatory) arrest**	**Check if NOT desired, to guide prehospital care for goals B and C** *(see reverse side)*
☐ Attempt CPR	☐ NO emergency intubation (goals B and C only)
☐ Do NOT attempt CPR	☐ NO assisted ventilation if unconscious (goal C only)

Explanatory notes on the discussion and instructions concerning specific interventions

Discussed with: ☐ User ☐ Representative | Name | Relationship

Contact information

Record the names of the participants as well as the words used during the discussion and all information that helps clarify the user's wishes.

Name of physician	Signature	Date (year, month, day)

Contact information

If a copy of this form is given to the user or his/her representative, it is signed by him/her so that paramedic ambulance technicians can follow the instructions on the form.

Name of user or representative	Signature	Date (year, month, day)

Explanatory notes

- **This form is not a substitute for consent to treatment, which must always be obtained *(except in certain emergency situations)*.**
- **This form must be signed by a physician.**

**Description of levels of care**
The discussion about levels of care is carried out with the user or, in the case of incapacity, with his/her representative, in the spirit of shared decision-making about medically appropriate care. The explanations and examples provided in the following descriptions do not assume capacity on the part of the user, nor do they necessarily reflect his/her usual care setting.

**Goal A**<br>**Prolong life with all necessary care**	• Care includes all interventions that are medically appropriate and transfer[1] if the intervention is not available in the current setting.<br>• All invasive interventions can be considered, including, for example, intubation and intensive care.
	▶ **In the prehospital setting**, unless otherwise advised by the user or his/her representative, all protocols apply; intubation, assisted ventilation[2] and assisted respiration[3] are included when appropriate.
**Goal B**<br>**Prolong life with some limitations to care**	• Care incorporates interventions with the aim of prolonging life, which offer the possibility of correcting deterioration in health status while preserving quality of life.<br>• Interventions may lead to discomfort that is judged to be acceptable by the user or his/her representative acting in the sole interests of the user, given the circumstances and the expected outcomes.<br>• Certain interventions are excluded since they are judged to be disproportionate[4] or unacceptable[4] by the user or his/her representative acting in the sole interests of the user, given the potential for recovery and undesired consequences *(e.g., short-term or long-term intubation, major surgery, transfer).*
	▶ **In the prehospital setting**, unless otherwise advised by the user or his/her representative, all protocols apply; assisted ventilation[2] and assisted respiration[3] are included; intubation is included unless indicated as not desired on the form (checked in the prehospital care box).
**Goal C**<br>**Ensure comfort as a priority over prolonging life**	• The user's comfort is prioritized through the management of symptoms.<br>• Interventions which may prolong life are used as needed in order to correct certain reversible health problems, by means judged acceptable by the user or his/her representative acting in the sole interests of the user *(e.g., oral or intravenous antibiotics to treat pneumonia).*<br>• Transfer to an appropriate care setting is considered only if care available locally is insufficient to ensure comfort *(e.g., for a hip fracture with significant discomfort or for respiratory distress at home).*
	▶ **In the prehospital setting**, unless otherwise advised by the user or his/her representative, all protocols apply; assisted respiration[3] is included; intubation and assisted ventilation[2] are included unless indicated as not desired on the form (checked in the prehospital care box).
**Goal D**<br>**Ensure comfort without prolonging life**	• Care is exclusively aimed at maintaining comfort through the management of symptoms *(e.g., pain, trouble breathing, constipation, anxiety).*<br>• Interventions do not aim to prolong life; illness is left to its natural course.<br>• A treatment that is usually given with curative intent may be used, but only because it represents the best option to relieve discomfort *(e.g., oral antibiotics for a lower urinary tract or C. difficile infection).*<br>• Transfer to an appropriate care setting is considered only if care available locally is insufficient to ensure comfort *(e.g., for a hip fracture with significant discomfort or for respiratory distress at home).*
	▶ **In the prehospital setting**, unless otherwise advised by the user or his/her representative, the following protocols apply: oxygenation, salbutamol, nitroglycerin (chest pain) and glucagon. For respiratory distress in a conscious user, assisted respiration[3] (CPAP) can be used unless refused. Intubation and assisted ventilation[2] are excluded. Manoeuvres to clear an obstructed airway in a living user can be carried out.

**Cardiopulmonary resuscitation (CPR)**
CPR is part of the same discussion as levels of care. The decision is specified in a distinct manner to allow rapid decisions in the case of cardiorespiratory arrest. A CPR decision is only applicable in the case of a cardiac arrest with arrest in circulation. In the case that a CPR attempt is desired, measures available on site will be deployed while awaiting the arrival of emergency medical services, according to the situation.

---

1 The term **"transfer"** implies moving the user to a setting that is different from his/her current environment (leaving his/her home, inter-institutional or intra-institutional transfer, etc.). If a transfer is not being considered, a care goal other than A must be selected.

2 **Assisted ventilation** is carried out via non-invasive techniques (bag-valve-mask, Oxylator) in an unconscious user.

3 **Assisted respiration** is carried out via non-invasive techniques (CPAP) in a conscious user.

4 The sense of the terms **"disproportionate"** or **"unacceptable"** is based on subjective perceptions and values that vary from person to person and across time. The words used by the user or his/her representative are important to record in the box provided for this purpose.
